# Cycling and Heart Disease

**Published:** 1895-10

**Authors:** 


### Cycling and Heart Disease.

Under the above caption, in the *Asclepiad*, No. 43, Vol. XI, 3d Quarter, 1894–'95, Sir B. W. Richardson publishes a revision of the interesting paper which he read before the Medical Society of London in January, on cycling in relation to diseases of the heart. The author has himself been a cyclist since 1877, and his powers of accurate observation and philosophic grasp are well known. What he has to say on this subject is therefore of great importance. The rapid increase in the number of persons using bicycles and the immoderation exhibited by some of them in the exercise, will unquestionably, before long, introduce among the inflammations, neuroses and muscular affections, cycler's cramp, cycler's heart, cycler's muscular strain, cycler's joints, etc.

Confining our attention to the effect of cycling upon the organs of circulation, Sir B. W. Richardson divides them into two classes: First, the immediate effects of the exercise upon the heart and circulation as observed on the rider. Second, the after-effects as observed in the consulting room or sick chamber.

First. In all riders, at all ages, in experts as well as beginners, there is in the beginning of each attempt a quickening of the circulation, although there may be no consciousness of the attendant phenomena. The pulse is full and bounding, and throughout the ride there is a continued rapidity not amounting to the same degree as at first, but rarely falling to less than one hundred pulsations per minute. The rise of the pulse is considerably increased in climbing, with a fall on horizontal planes and a well-marked fall in descents, especially if the feet be taken off the pedals, as is the practice of accomplished cyclists. Even if cycling be daily continued, these phenomena will be excited. The heart, if examined during a few moments of rest, in order to permit of auscultation, is full and bounding like the pulse. The external impulse is very pronounced, and the sounds are full with not unfrequently an accentuation of the second sound. So long as the exercise is continued, an increase of cardiac motion is observable, the act of movement on the machine seeming sufficient to keep the circulation in vigorous and equal tension. This accounts, according to Richardson, for the astounding journeys that the fully trained cyclist can undertake, when in his prime, and for his endurance against sleep. There are some peculiar points connected with this overaction of the heart. For example, no rider is so embarrassed by it as to cause him to stop abruptly in order to dismount and seek rest, while one rider, who could not climb a flight of stairs on foot without resting many times during the ascent, complaining of breathlessness and palpitation, could, on the machine, climb hills without distress. It would be wrong to conclude from this that cycling is not injurious, because there has not been length of time enough to determine from many cases what the ultimate effect of long continued riding may be. The evidence on this particular subject is unfavorable at a general glance, for several accomplished and skillful riders have, after some years, succumbed prematurely from diseases of the circulation, but there has been no sufficient pathologic inquiry to prove in what way the damage was developed.

Second. Dr. Petit suggests that out of one hundred riders there is sure to be one at least who is affected with heart disease. The wonder, therefore, is why so few suffer in an immediate manner from the exercise. Petit seems to have known of two or three sudden deaths, but he does not tell how many hundreds or thousands of persons form the body of riders, out of which this conclusion was drawn. Richardson has been giving attention to the matter since 1887, and knows of only five or six instances, physical accidents excluded, in which a cyclist is said to have died during the exercise, and he is not sure that in any of these cases the fatal result was to be attributed to the influence of the exercise upon the heart. If, however, we have to consider the continuous effect for some years on those in whom the elastic tissues have lost much of their primal elasticity, it is certain that there are many men and women, in whom the circulation becomes disturbed ("distrained" is Richardson's word) by an arduous pursuit of the exercise. Fortunately there comes with this a "saving" distaste for the exercise which gives protection. For some obscure reason, one who has been a cyclist gives up using his wheel. Upon examination it is found that there is a feebleness of the circulation, coldness of the extremities and an unnatural languor and inability to sustain fatigue and a rather quick weariness if exercise on the machine be tried

Contrary to what would be expected theoretically, cycling exercise carried out with moderation two or three times a week, if it be done without strain, as in hill climbing, and if it be not too long continued, as in a long stretch, proves an actual remedy in cases of fatty degeneration of the heart. Richardson relates, indeed, a case in which the exercise proved beneficial to a man of over seventy-five years, suffering with symptoms of senile failure of the heart. Horse exercise he believes not in the least degree comparable with cycling in these cases, while walking in any degree is all but impossible, because the limbs have to carry the weight of the trunk, and fatigue, which is very wearing, leads to more exhaustion than is balanced by the exercise.

Gouty dyspepsia is often very much benefited by moderate cycling. In cases of marked valvular disease, the exercise is not to be advised, but there are some cases in which it has been undertaken without apparently resulting harmfully. Intermittent pulse and palpitation may be improved by exercise on the tricycle rather than the bicycle, so that the patient may at any moment stop without alighting, and shall not undergo the nervous strain which attends bicycling. In anemia, the exercise may be directly curative, especially in the case of women.

Overstrain in cycling is not merely a theoretic danger but has actually been observed. There are two classes of subjects who are affected injuriously. The first are young persons, often mere boys, who are made to ply the machine, probably heavily loaded, for commercial duties and business. The boy really does the work of a horse in this way; he seems to enjoy it, and the employers, knowing no evll from it, let him do all that may be done. On account of the immaturity of the heart and arteries, they are easily expanded under improper pressure, and cardiac hypertrophy and disproportionate development of the heart and lungs is the result. Secondly, there are the extreme conditions shown in those remarkable athletes, who enter into competitions that have never before been dreamed of in the history of the world. The heart of the cyclist accomplishes in twenty-four hours a labor equal to lifting one hundred tons one foot from the earth, and this without sleep or rest on the part of the rider. Such feats can not be repeated many times by one person without mischief to the heart. As a matter of fact, Sir B. W. Richardson has seen many cases, even among the so-called best athletes, in which the heart has become large, irritable, extra sensitive and easily intermittent. The arteries are distended, their elastic tissues enfeebled, and their functions as regards nutritive repairs imperfect. In both these classes of cases, the young boys who are made to work too hard, and the athletes who engage in extravagant competion, degenerative change in the organs of the body generally is a result of the injury done to the heart and arteries. In advising patients on the subject of cycling, it is often more important to consider the state of the vessels than that of the heart. Enfeebled and worn out arteries are more dangerous than an enfeebled heart.

There are three sets of acts which are most injurious in cycling; these are straining to climb hills or to meet head winds, excessive fatigue, and the process of exciting the heart and wearing it out sooner by alcoholic stimulants, to the omission of light, frequently repeated and judiciously selected food.—*Philadelphia Polyclinic.*

